# Dental News Letter

**Published:** 1858-11

**Authors:** 


*The Dental Reporter*, for August, contains much that will interest, in addition to condensed reports of the proceedings of the "Western Dental Society," and of the "American Dental Convention;" but we are sorry to see in it a paper which we can not endorse, as it advocates a principle entirely repugnant to our feelings and convictions of right, and against which we must enter a protest. It is entitled "Extracting Teeth by Galvanism," signed "Jerome."

The writer, after giving the method of applying the agent, and reports of cases—as have been previously published—with descriptions of various kinds of batteries, winds up in the following extravagant spread-eagle style:—

"We learn that many are experimenting regardless of the patent, and we think the owners of the patent will find that they are engaged in rather an unprofitable business. They may annoy some, but nine-tenths of the profession will use it without a right—*except that right which Almighty God and the spirit of our Republican institutions grants to every man—the pursuit of happiness, the acquisition of knowledge, and the use of such knowledge.*"

The sublimity and completeness of the italicized sentence (the italics are ours) would be greatly enhanced, we think, by the following amendments:—After the name of the Deity, (which we greatly regret to see in such connection), add the words "the Continental Congress." After the words "Republican Institutions, add, "and the Genius of Filibusterism;" and at the close of the sentence, add that emphatic and popular endorsement, "That's so."

But, seriously, we submit to the intelligent reader, whether this same spirit of "*acquisition*," though exhibited in other forms, has not interfered materially with the liberty of the "*pursuit of happiness*" of many a man, by confining him in one of a certain class of *public institutions*. Notwithstanding we may sincerely regret and strongly discourage (as we do regret and discourage) this disposition to patent every thing, still we must condemn such sentiments as the above, as erroneous and unjust in principle—in appropriating, without regard to ownership, that which the law recognizes and protects as property—and this we do without any reference whatever to the particular case under discussion, but *on the abstract principle involved.*

J. R. M'C.

The foregoing is copied from the *Dental News Letter*, for October.

The exceedingly pious junior Editor of the Dental News Letter seems much shocked, that the name of the Deity should be used in connection with such a subject, probably on the same ground that a pious Jew, when questioned in relation to his grasping in business, replied, "Why, what has religion to do with business?" I would ask, why should *He*, "who holds the *Lightning in his hand*," not be mentioned in this connection? Does my friend deem the power of a patent higher or stronger than that of Almighty God? For my part, I am neither afraid nor ashamed to confess to the power and wisdom of God, and to look to Him for knowledge and strength, in every act and undertaking.

The suggestions made by my friend, J. R. M'C., are doubtless such as are suited to *his* tastes and inclinations, and if so, I have not the least objection to his adoption of them.

It is news to me, that the spirit of acquisition, authorized by God and the spirit of our institutions, is such as to be deemed felonious; if, as is assumed, the patent laws stand in opposition to these greater and more sovereign rights, it is high time they were repealed or amended.

"But seriously," as remarks my friend, I submit to the intelligent reader, whether, in my article above referred to, one word, sentence, or paragraph, can be found, advising, encouraging, or prompting to the subversion of any law, right, or privilege. I stated what I deemed a simple fact, which I believe has been fully verified by the experience of every man familiar with the history of Dental patents. *I have not made* it so, but *it exists*, and Mr. McCurdy himself can not but acknowledge, that his own experience in the subject under consideration, fairly confirms my prediction; else, why does he sell so many more Batteries without a patent, than he sells with a patent right?

While I am as much opposed to the infringement or violation of any just right or claim, as Mr. McCurdy can or dare be, I can not shut my eyes to the facts, as illustrated by almost every Dental patent ever issued. There has, from the beginning, been a question, whether the Francis Patent was valid or no; and every day but strengthens the opinion that it is not.

What, then, does all this flourish of high-sounding names, quotations and *Italics* mean. He seems, like the ancient war-horse, to smell the battle from afar, and immediately commences to rear, and pitch, and snort, to attract attention to the simple fact, that he has discovered a "mare's nest;" or, like a "blinded bruiser," he "fights wild," hoping to strike some person; but if he has, by accident or otherwise, hit any one, I

must confess my inability to discover who or where it is, unless, like the Benecia boy, he struck the *stake* instead of his man!

Take heed, friend Mac, and do not let your passions and prejudices get the better of your perceptions and judgment, nor your love of notoriety lead you into an attack upon shadows; wait for the substance, and your readers will be edified by your discretion.

Mr. McCurdy condemns *sentements*, and points to my article in the "Reporter" for them, where they do not exist at all.

I stated a simple fact, which the intelligence of every dentist will confirm,—that nine-tenths of the profession will use the electrical process of extracting teeth, without a patent right. I did not say that it was *right*, nor that it was *wrong*, but simply that such *would be* the result.

And I now appeal to the Profession to attest, whether or not my prediction was correct. I know, of my own knowledge, and I am sure others who have had as much opportunity for observation can not but know, that at least nine-tenths of those who use an Electro-Galvanic Battery for extracting teeth, do so without any patent right at all. "That's so."

JEROME.

